# Next‐Generation Vitrimers Design through Theoretical Understanding and Computational Simulations

**DOI:** 10.1002/advs.202302816

**Published:** 2023-12-07

**Authors:** Ke Li, Nam Van Tran, Yuqing Pan, Sheng Wang, Zhicheng Jin, Guoliang Chen, Shuzhou Li, Jianwei Zheng, Xian Jun Loh, Zibiao Li

**Affiliations:** ^1^ Institute of Materials Research and Engineering (IMRE) Agency for Science, Technology and Research (A*STAR) 2 Fusionopolis Way, Innovis #08‐03 Singapore 138634 Republic of Singapore; ^2^ School of Materials Science and Engineering Nanyang Technological University 50 Nanyang Avenue Singapore 639798 Singapore; ^3^ Institute of Sustainability for Chemicals, Energy and Environment (ISCE2) Agency for Science, Technology and Research (A*STAR) Singapore 138634 Singapore; ^4^ Laboratory for Biomaterials and Drug Delivery The Department of Anesthesiology Critical Care and Pain Medicine Boston Children's Hospital Harvard Medical School Boston MA 02115 USA; ^5^ Institute of High Performance Computing (IHPC) Agency for Science, Technology and Research (A*STAR) 1 Fusionopolis Way, #16‐16 Connexis Singapore 138632 Republic of Singapore; ^6^ Department of Materials Science and Engineering National University of Singapore Singapore 117576 Singapore

**Keywords:** bond exchange reactions, density functional theory, molecular dynamics simulations, Monte Carlo simulations, vitrimers

## Abstract

Vitrimers are an innovative class of polymers that boast a remarkable fusion of mechanical and dynamic features, complemented by the added benefit of end‐of‐life recyclability. This extraordinary blend of properties makes them highly attractive for a variety of applications, such as the automotive sector, soft robotics, and the aerospace industry. At their core, vitrimer materials consist of crosslinked covalent networks that have the ability to dynamically reorganize in response to external factors, including temperature changes, pressure variations, or shifts in pH levels. In this review, the aim is to delve into the latest advancements in the theoretical understanding and computational design of vitrimers. The review begins by offering an overview of the fundamental principles that underlie the behavior of these materials, encompassing their structures, dynamic behavior, and reaction mechanisms. Subsequently, recent progress in the computational design of vitrimers is explored, with a focus on the employment of molecular dynamics (MD)/Monte Carlo (MC) simulations and density functional theory (DFT) calculations. Last, the existing challenges and prospective directions for this field are critically analyzed, emphasizing the necessity for additional theoretical and computational advancements, coupled with experimental validation.

## Graphical abstract

MD/MC simulations elucidate the intricate relationships among structure, dynamics, and properties, enhancing comprehension of how polymer chain behaviors impact relevant properties. They provide insights into tailoring vitrimer properties on a macro scale. Concurrently, DFT calculations delve deeper into chemical nuances, aiding in the design of bond exchange mechanisms and fine‐tuning reaction kinetics.

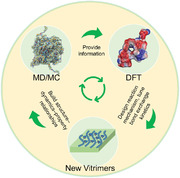

## 1 Introduction

Plastics have emerged as one of the most pivotal materials in the past century, significantly contributing to advancements across various domains.^[^ ^1^ ^]^ Primarily, plastics can be divided into two categories based on the molecular arrangement of their chains, namely thermoplastics and thermosets. In thermoplastics, the chains are not covalently linked together, making the material behave as a viscoelastic liquid upon heating. Thermoplastics can be recycled repeatedly but lack mechanical and chemical resistance due to the absence of cross‐linking. Thermosets, on the other hand, are formed by covalently cross‐linking the polymer chains, resulting in a 3D network that exhibits high mechanical strength and solvent resistance. However, thermosets are not recyclable due to their irreversible cross‐linking nature. Therefore, the challenge lies in developing a high‐performance, recyclable polymeric material that combines the advantages of both thermoplastics and thermosets.

The development of covalent adaptable networks (CANs) represents a notable advancement toward achieving such materials. CANs are a new class of polymeric materials that have garnered considerable attention as an intriguing category of sustainable plastics, owing to their potential to display strength, durability, and chemical resistance compared to conventional thermosets, coupled with the added advantage of recyclability at end‐of‐life.^[^ ^2^ ^]^ Unlike traditional covalent network polymers, CANs possess a dynamic network that can be reconfigured or adapted by chemical reactions without compromising their mechanical stability.^[^ ^2b^ ^]^ This characteristic is due to the presence of exchangeable covalent bonds, which can be broken and reformed reversibly under appropriate stimuli, such as heat, catalyst, light, and pH.^[^ ^2^, ^3^ ^]^ In the absence of these stimuli, the cross‐linked structure of CANs endows them with thermoset‐like properties such as high strength and durability. Importantly, unlike traditional thermosets, which are essentially non‐recyclable, CANs can in principle be recycled due to the dynamic nature of their crosslinks.^[^ ^2^, ^4^ ^]^

CANs are an emerging class of materials, primarily classified into dissociative and associative networks based on their bond exchange mechanisms.^[^ ^3d,b^ ^]^ Dissociative networks involve dynamic bond cleavage, followed by new covalent bond formation, leading to a decrease in crosslinking density, enabling stress relaxation and flow. Associative networks, conversely, maintain a constant crosslink density through associative exchange mechanisms. Vitrimers were first introduced in 2011 by Leibler and colleagues, who noted that these materials exhibit a viscosity–temperature relationship akin to that of vitreous silica, characterized by an Arrhenius‐like dependence.^[^ ^2h^ ^]^ In vitrimers, the associative exchange process results in a constant crosslinking density that remains unchanged with time or temperature.^[^ ^3^, ^5^ ^]^ These materials display the capacity to flow and undergo processing above a specific temperature threshold; while, exhibiting thermoset‐like behavior below it. Further, the temperature dependence of the viscosity can be regulated by manipulating the rates of chemical exchange reactions.^[^ ^5a^ ^]^

Since the advent of the vitrimer concept, numerous dynamic covalent bonds have been utilized to construct cross‐linking networks, encompassing transesterification, silyl ether,^[^ ^6^ ^]^ vinylogous urethane,^[^ ^7^ ^]^ imine bond,^[^ ^8^ ^]^ urethane/urea,^[^ ^9^ ^]^ boronic ester,^[^ ^10^ ^]^ disulfide,^[^ ^11^ ^]^ olefin metathesis,^[^ ^12^ ^]^ and so on. Contemporary investigations on vitrimers are primarily concerned with understanding their fundamental properties,^[^ ^5^, ^13^ ^]^ advancing their synthesis and processing techniques,^[^ ^3^, ^14^ ^]^ enhancing their self‐healing and recyclability attributes.^[^ ^15^ ^]^ The prospective applications of vitrimers are broad, encompassing diverse fields such as soft robots, functionally tunable devices, car industries, and aerospace.^[^ ^3^, ^16^, ^58^ ^]^ However, the mechanical performance of vitrimers, particularly their ultimate tensile strength, remains inadequate when compared to the capabilities of conventional industrial thermoplastic and thermoset.^[^ ^17^ ^]^ The structure–property relationships in vitrimers play a pivotal role in their performance and applications, necessitating a thorough comprehension of these materials. Factors including the polymer backbone and dynamic bond types, crosslink density, microphase clusters, and network architecture dictate these relationships.^[^ ^17^, ^18^ ^]^ By meticulously controlling these parameters, researchers can tailor vitrimers properties to satisfy the demands of diverse applications. For example, augmenting the cross‐linking density can bolster mechanical strength; while, incorporating catalysts can modulate bond exchange rates, and consequently, the material's responsiveness to external stimuli.^[^ ^16^, ^19^ ^]^ Developing a clear understanding of the relationship among vitrimers’ structure, dynamics, and performance is crucial for designing vitrimers with targeted properties. This understanding allows researchers and engineers to tailor vitrimers for specific applications by manipulating various aspects of their composition and processing conditions.

Computer simulations have played a crucial role in understanding the structure, dynamics, and performance of vitrimers and have provided insight into the design and synthesis of new materials. In this review paper, we focus on the use of various computational methods, including density functional theory (DFT), molecular dynamics (MD), and Monte Carlo (MC), to investigate the properties of vitrimers. The key feature of vitrimers is their ability to undergo exchange reactions while maintaining their network topology, which makes them adaptable and recyclable. DFT serves as a crucial instrument for comprehending the relationship between structures and performances.^[^ ^7^, ^20^ ^]^ This process entails the development of a rational reaction model to investigate the elementary steps of the reaction and pinpoint the key factors influencing the reaction rate, subsequently optimizing the chemical reaction conditions. Consequently, theoretical calculations aid in discerning the fundamental process and reaction pathway at the atomic level. MD simulations and MC simulations have emerged as powerful tools to investigate the behavior of vitrimers at the atomic level, providing insights into their dynamics, structure, and properties.^[^ ^21^ ^]^ MD can model fast dynamics at the atomic scale^[^ ^22^ ^]^ and MC can sample larger conformational spaces and explore thermodynamic properties.^[^ ^23^ ^]^ This hybrid approach allows for efficient and accurate simulations of vitrimers, which exhibits both fast and slow dynamics, as well as complex phase behavior.

**Figure 1:**
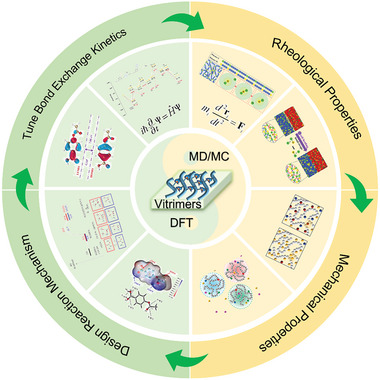
Scheme and image illustration of computational understanding and aid design vitrimer materials. Reproduced with permission.^[^ ^21c^ ^]^ Copyright 2022, American Chemical Society. Reproduced with permission.^[^ ^24^ ^]^ Copyright 2020, PNAS. Reproduced with permission.^[^ ^21a^ ^]^ Copyright 2022, American Chemical Society. Reproduced with permission.^[^ ^20k^ ^]^ Copyright 2022, Wiley. Reproduced with permission.^[^ ^25^ ^]^ Copyright 2022, Elsevier. Reproduced with permission.^[^ ^20a^ ^]^ Copyright 2022, American Chemical Society.

This review article aims to comprehensively summarize recent computational studies on vitrimer materials, with particular emphasis on understanding the underlying principles governing their behavior and guiding the design of new materials (**Figure** **1**). Specifically, we address the question of how computational simulations can contribute to the study of vitrimers and summarize and discuss representative studies from the past decade. In Section 2, we discuss the different computational methods that have been used to study vitrimers, including DFT, MD, and MC. We also highlight the potential for machine learning techniques to enhance the efficiency and accuracy of computational design approaches In Section 3, we explore computational research on vitrimers, offering molecular insights into their behavior. We examine studies focused on dynamics, bond exchange reactions (BERs), and versatile vitrimer properties. Further, we outline how computational simulations can pave the way for designing innovative vitrimers. This encompasses discussions on employing MD/MC simulations to tune bond exchange kinetics, optimize crosslinking chemistry, predict material properties, and utilize DFT calculations for pre‐screening and functional group modification prior to experimental synthesis. Overall, this review demonstrates the significant role that computational simulations can play in advancing our understanding of vitrimeric materials and facilitating the development of new materials with desirable properties for various applications.

## 2 Simulation Method Overview

### 2.1 Density Functional Theory (DFT)

DFT is a quantum mechanical framework primarily used to investigate the electronic structure of many‐body systems, particularly atoms, molecules, and solids. It is predicated on the principle that the ground state properties of a quantum system can be determined through its electron density alone, rather than its wave function, making it computationally more tractable for larger systems.

**Figure 2:**
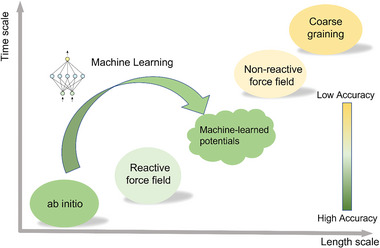
Simulation methods. Computational accuracy versus simulation time and length scale. MLMD emerging as a potential approach to address the balance between computational cost and accuracy in conventional MD simulations.

In terms of vitrimers, DFT can be used to perform geometry optimization and calculate the electronic properties of vitrimers, such as electron density,^[^ ^26^ ^]^ which then can be related to the covalent bonds formation/dissociation in the material, allowing for a deeper understanding of the relationship between the electronic structure and the bonding exchange of the material. DFT can also be used to obtain the thermodynamic and kinetic properties, which involves breaking and reforming the dynamic covalent bonds.^[^ ^27^ ^]^ Transition state search techniques such as nudged elastic band^[^ ^28^ ^]^ or climbing image‐nudged elastic band^[^ ^29^ ^]^ are often employed in DFT calculations to locate the transition state of a chemical reaction, which corresponds to the highest energy point along the reaction pathway. This technique plays a crucial role in elucidating reaction mechanisms and understanding the energetics of chemical transformations. By calculating these processes, researchers can gain insight into the factors that influence the efficiency of reprocessing, such as temperature, catalyst, and more. Moreover, DFT serves as a valuable approach for obtaining the potential employed in molecular dynamic simulations, a topic that will be comprehensively addressed in the subsequent section. Although DFT can provide very high accurate results, the extremely high computational cost of the electronic structure calculations limits the size of the considered systems (**Figure** **2**). Second, the static DFT calculation cannot account for the dynamics of the polymer under different working conditions, which is very important to study their working mechanisms.

### 2.2 Molecular Dynamics (MD) Simulation

MD is a widely used technique for studying the dynamical behavior of a classical many‐body system.^[^ ^30^ ^]^ This approach enables the examination of the spatiotemporal evolution of a many‐body system, typically through the numerical solution of Newton's Equations of motion for a group of interacting particles, wherein the trajectories of atoms and molecules are determined.

Once the initial particle coordinates and velocities, along with the corresponding potential energies are established, the temporal evolution of atom position and velocity can be calculated. An essential aspect in the realm of MD simulations lies in the acquisition of the potential energy. Diverse methodologies have been used for the derivation of this potential, including approaches rooted in ab initio calculations (commonly recognized as ab initio molecular dynamics [AIMD]) or those relying on force fields (including both classical force fields and reactive force fields). Recently, there has been a trend toward employing machine‐learned potentials (MLPs) for approximating the potential energy. The general concept, advantages, and disadvantages of each approach in simulating vitrimers will be discussed below.

#### 2.2.1 Ab Initio Molecular Dynamics (AIMD)

The AIMD method is a computational approach that integrates quantum mechanical calculations with MD simulations. In AIMD, the interactions among atoms are described using first‐principles methods, typically based on DFT as mentioned in the previous section, which accurately captures the electronic structure and energetic properties of molecules and materials. As AIMD directly solves the electronic Schrödinger equation for a system, it could take into account the quantum behavior of electrons and their interactions with atomic nuclei. This leads to more accurate descriptions of chemical reactions, bond breaking and formation, and other electronic processes. Therefore, AIMD can provide insights into reaction mechanisms, molecular conformations, and the behavior of materials under various conditions, such as temperature and pressure. However, AIMD simulation is computationally intensive, which limits its use in simulating polymer systems, including vitrimers. Consequently, research using AIMD in this field is scarce.

#### 2.2.2 Classical Molecular Dynamics (CMD)

In CMD simulations, the potential energy functions are delineated using force fields. These force fields comprise a series of mathematical formulations coupled with corresponding parameters, tailored to compute the potential energy of a particle system, typically encompassing atoms or molecules. Due to their simpler form of mathematical functions, interatomic potentials are more computationally efficient than quantum methods such as DFT, allowing for the investigation of larger systems of atoms. This computational advantage makes it possible to use CMD simulations to model vitrimers, overcoming the system size limitations of DFT. In addition, combining CMD with coarse‐grained (CG) models where a group of atoms is conceptualized as a single particle or bead, referred to as CGMD, further increases the system size that can be studied (Figure 2).^[^ ^21^, ^31^ ^]^ As a result, MD simulations have become a widely used technique in studying vitrimers. Over the last few decades, MD simulations have become the dominant technique in the field of computational simulation of polymers.^[^ ^23^, ^32^ ^]^ This is largely due to the dynamic nature of the technique and its ability to provide real‐time information about the system evolution, which cannot be obtained by any other method.

However, the method itself also poses some challenges. Despite the availability of many force fields, the universality and accuracy of each often conflict with one another.^[^ ^33^ ^]^ This challenge is inherent to the method and has led to the development of force fields that focus on specific areas, which can make their transferability to other systems/areas problematic.^[^ ^34^ ^]^ Consequently, the selection of appropriate force fields and models is crucial to achieving reasonable results in MD simulations. Classical force fields such as AMBER,^[^ ^35^ ^]^ GAFF,^[^ ^36^ ^]^ CHARMM,^[^ ^37^ ^]^ GROMOS,^[^ ^38^ ^]^ OPLS,^[^ ^39^ ^]^ DREIDING,^[^ ^40^ ^]^ MMFF94,^[^ ^41^ ^]^ UFF,^[^ ^42^ ^]^ COMPASS,^[^ ^43^ ^]^ PCFF,^[^ ^44^ ^]^ and others are frequently used to study polymer systems.

Most force fields employ harmonic oscillators to model bond interactions. However, these models have limitations when simulating chemical reactions as they fail to account for bond formation and breaking when the distance between two bonded atoms becomes infinitely large. This drawback hinders their effectiveness in probing chemical reactions, a crucial process in vitrimer systems. A solution for this problem is to provide a mechanism that performs bonding swap between polymer chains.^[^ ^31^, ^45^ ^]^

A more realistic method to determine bonding patterns could be through the use of a reactive force field. Unlike classical force fields, reactive force fields can provide a more accurate representation of chemical reactions and dynamic processes, allowing them to capture bond‐breaking and bond‐forming events during chemical transformations. Therefore, these force fields are particularly suited for simulating reactions involving bond rearrangements, radical species, and transition states. ReaxFF,^[^ ^46^ ^]^ Tersoff,^[^ ^47^ ^]^ REBO,^[^ ^48^ ^]^ Brenner,^[^ ^49^ ^]^ and APT^[^ ^50^ ^]^ are among the most popular reactive force fields used to study reactions in solids, liquids, and gases. To simulate the bond formation/dissociation, these force fields take into account the local environment (bond order) to describe interatomic interactions. As said, the strength of bonds is modified by the environment of the atom allowing them to form/break during the simulation. However, these force fields are often limited in their application to specific systems, require more computational resources, and are challenging to parameterize. Recently, machine learning methods based on neural networks have been applied to train ReaxFF to raise the simulation accuracy as well as increase the simulation speed via using large MD timesteps.^[^ ^51^ ^]^

#### 2.2.3 Hybrid Molecular Dynamics (MD)–Monte Carlo (MC) Simulation

The MC simulation is a quantitative method that offers probabilistic estimates for outcomes of indeterminate events.^[^ ^52^ ^]^ Generally, this method generates a new system configuration by randomly selecting and moving a molecule; and then, accepting or rejecting the move based on a suitable acceptance rule. The ensemble of configurations produced using this technique can be used to calculate the thermodynamic and structural properties of the system given certain conditions. To determine the dynamic properties of the bulk vitrimer in several physical states, hybrid MD–MC algorithms are often used to study the bulk vitrimer system.^[^ ^21^, ^53^ ^]^ The MD‐MC simulation approach combines the strengths of both MD and MC simulations to study molecular systems. In this framework, the MD component integrates the Newtonian equations of motion, capturing the inherent dynamics and consequently elucidating the flexibility of the vitrimer. Concurrently, the MC segment is instrumental in characterizing the bond exchange behavior, thereby facilitating the modulation of dynamic bonds. Combining MD and MC can overcome the limitations faced by each method individually. For example, MC might be able to determine the bond exchange behavior in vitrimer that MD struggles with, and MD can provide detailed trajectories that MC lacks. The MD–MC simulation approach also has its own set of disadvantages.^[^ ^54^ ^]^ For example, the hybrid approach can be computationally expensive because it essentially couples two distinct simulation methods, requiring more computational resources and time.

#### 2.2.4 Machine Learning Molecular Dynamics (MLMD)

An alternative method for simulating the formation and breaking of dynamic bonds in molecular simulation is the utilization of MLMD.^[^ ^55^ ^]^ This approach employs machine learning algorithms to construct an accurate representation of interatomic interactions for MD simulations. MLMD is an attractive option because it is computationally more affordable than AIMD; while, still maintaining accuracy and cost‐effectiveness (see Figure 2).^[^ ^56^ ^]^ As a result, the technique can bridge the gap between AIMD and CMD simulations. Typically, MLPs are obtained by fitting energy and force data generated from DFT or AIMD calculations.^[^ ^56^ ^]^ MLMD has gained a lot of attention in recent years, as evidenced by the rapidly increasing number of developed MLPs, such as Behler–Parinello Neural Networks,^[^ ^57^ ^]^ q‐SNAP,^[^ ^58^ ^]^ GAP potentials,^[^ ^59^ ^]^ and others.^[^ ^56^ ^]^ Together, with the development of MLPs, there are also several tools and packages developed to help design and train those MLPs such as DeePMD‐kit,^[^ ^60^ ^]^ PiNN,^[^ ^61^ ^]^ SchetPack,^[^ ^62^ ^]^ TensorMol,^[^ ^63^ ^]^ aenet,^[^ ^64^ ^]^ MLatom,^[^ ^65^ ^]^ AMP,^[^ ^66^ ^]^ PROPHet,^[^ ^67^ ^]^ TorchANI,^[^ ^68^ ^]^ DScribe,^[^ ^69^ ^]^ ChemML,^[^ ^70^ ^]^ and SimpleNN.^[^ ^71^ ^]^

However, the advancement of MLPs also encounters a series of challenges.^[^ ^55^, ^72^ ^]^ Foremost among these is the task of generating an extensive and high‐quality dataset for training purposes. MLPs require substantial high‐quality training data to accurately learn the potential energy landscape. Obtaining diverse and representative data (normally though expensive DFT calculations) that covers a wide range of chemical environments and configurations can be challenging. In addition, data bias can be a big problem. If the training data is biased toward specific chemical structures or energy landscapes, the MLP may exhibit inaccuracies or limited generalization capabilities, leading to poor performance. The second issue is to improve the transferability. Ensuring the transferability of MLPs across different systems, conditions, and phases (e.g., gas, liquid, and solid) remains a significant challenge. An MLP trained for one specific system might not perform well for different systems or conditions. In addition, achieving chemical accuracy with MLPs is demanding. While MLPs can accurately predict energetics and forces, capturing fine electronic details such as charge redistribution and polarization effects may be difficult. Finally, the training of MLPs can be computationally intensive, requiring substantial time and resources.

## 3 Molecular Insights and Aided Design of Novel Vitrimers

Acquiring molecular insights into vitrimers is crucial because it facilitates comprehension of their distinctive characteristics and behavior. By scrutinizing the structure of vitrimers, researchers can enhance their understanding of the molecular‐level mechanisms that underlie the behavior and properties of these materials. This, in turn, can pave the way for the development of novel and improved vitrimer materials. Moreover, comprehending the structure of vitrimers can enable the optimization of their properties for specific applications, including the production of adhesives, coatings, and composites.^[^ ^73^ ^]^ Despite significant advancements, the complex dynamic behavior demonstrated by vitrimers remains not entirely understood, particularly at the molecular level, due to the novelty and intricacy of these materials.^[^ ^74^ ^]^ The dynamic rearrangement of the topological network and the capacity to externally control the rate of exchange reactions of vitrimers are crucial factors in understanding the intricate structure and predicting the properties of these systems. Consequently, there has been significant effort in simulating the dynamics of vitrimer systems. Two common approaches to polymer simulations of vitrimers are the “atomistic” and “CG” methods. Although the atomistic approach facilitates the investigation of specific polymeric chemistries, its high level of detail presents challenges when exploring large systems or extended timescales. This limitation becomes particularly restrictive for polymer networks in which overall structural relaxation times substantially increase. Conversely, the CG approach employs simplified polymer models to decrease computational complexity while preserving crucial physical interactions between monomers. Consequently, the CG approach enables the examination of larger systems and longer timescales in comparison to the atomistic approach.

### 3.1 Representation of Dynamic Bond Exchange

The primary complexity in vitrimers simulation arises from temperature‐sensitive reversible cross‐link reactions that dynamically modulate their mechanical behavior.^[^ ^75^ ^]^ The majority of force fields utilize harmonic oscillators to characterize bond interactions. Nevertheless, such models encounter challenges in accurately representing chemical reactions. This limitation curtails their aptitude for examining chemical reactions, an indispensable aspect of vitrimer systems. A viable remedy to this challenge is the incorporation of a mechanism that facilitates bond exchanges between polymer chains (**Table** **1**).

**Table 1:** A summary of the representative computational methods for vitrimer and the corresponding system size.

Method	System size	Ref.
“Cut‐off” distance approach (atomistic MD)	≈10^0^–10^1^ nm ≈10^1^–10^2^ ns	[21, 76]
Accelerated ReaxFF (atomistic MD)	≈10^0^–10^1^ nm ≈10^1^ ps	[73, 77]
Three‐body potential (CGMD)	≈10^1^–10^2^ nm ≈10^1^ µs	[21, 45]
Hybrid CGMD‐MC	≈10^1^–10^2^ nm ≈10^7^ timestep	[21, 24, 53]

**Figure 3:**
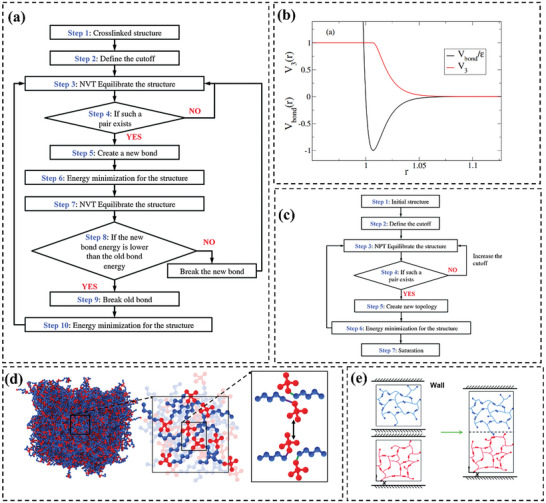
a) Flowchart of the bond exchange reaction process. Reproduced with permission.^[^ ^76a^ ^]^ Copyright 2015, Royal Society of Chemistry. b) The radial dependence of a (normalized) typical bond potential and the associated V_3_(r) potential. Reproduced with permission.^[^ ^21f^ ^]^ Copyright 2017, Springer. c) Flowchart of the crosslink process. Reproduced with permission.^[^ ^76a^ ^]^ Copyright 2015, Royal Society of Chemistry. d) 3D coarse‐grained network of simulated vitrimer. Reproduced with permission.^[^ ^21i^ ^]^ Copyright 2023, Wiley. e) Schematic graphs of the MD simulation of the welding process. Reproduced with permission.^[^ ^83^ ^]^ Copyright 2016, Royal Society of Chemistry.

These techniques allow MD simulations to investigate bond exchange reactions of vitrimers. For example, Yang et al.^[^ ^76a^ ^]^ overcame this issue by implementing a distance‐based reaction cutoff, which greatly accelerated the chemical dynamics (**Figure** **3**a). Bonds were created based on the proximity of reacting atoms and the topology was accepted based on the energy of the new bond. The results of their study successfully demonstrate the capability of the atomistic MD approach in capturing the network rearrangement behavior of vitrimers. With the new MD procedure, they were able to monitor the movement of active atoms during a sequence of BERs. Singh et al.^[^ ^78^ ^]^ further improved the method by employing an explicit temperature dependence of reaction probabilities drawn from experimental insights. Further, Park et al.^[^ ^73a^ ^]^ formulated a disulfide‐type BER algorithm, wherein bond breaking and formation occurred based on probability to capture the bond exchange chemistry in atomistic MD simulations. The aforementioned “cut‐off distance approach” possesses certain limitations as it assesses the probability of reactive sites engaging in a reaction based solely on their relative distance, without taking into account the reaction pathway. One solution to realistically account for the bond exchange reaction is to use the same kind of reactive force fields such as ReaxFF. For example, Kamble et al.^[^ ^73b^ ^]^ employed the ReaxFF method in conjunction with the bond boost approach^[^ ^79^ ^]^ to model carbon‐fiber reinforced vitrimer composites. This approach enabled the simulation of exchange reactions under realistic low‐temperature conditions, thereby mimicking experimentally observed chemical reactions and mitigating the occurrence of undesired side reactions associated with high temperatures.

However, the intensive computational demands of all‐atom simulations restrict system size and accessible time scales (Table 1), impeding accurate depiction of intricate dynamics in vitrimers. Coarse graining offers a feasible solution, streamlining system representation and enhancing simulation efficiency, thereby aligning molecular modeling closer to experimental time scales. Smallenburg and coworkers.^[^ ^31^ ^]^ introduced a simple patchy particle model system to study the phase behavior and dynamics of vitrimers. The research employed event‐driven molecular dynamic simulations and Wertheim's theory for free energy estimations in vitrimer modeling.^[^ ^80^ ^]^ Utilizing the Kern–Frenkel model,^[^ ^81^ ^]^ designed particles ensure exclusive single‐bond patch interactions, fulfilling the vitrimer network's associative bond exchange criteria. The research team further improved the method by introducing a three‐body potential in conjunction with thermodynamic perturbation theory to mimic the bond exchange mechanism in vitrimers.^[^ ^23e,f^ ^]^ The foundational concept of the three‐body potential hinges on the incorporation of a repulsive potential (see Equation (1) and Figure 3b) arises from tripartite interactions among particles *i–j* and *i–k*. This formulation also integrates a parameter *λ* to navigate between bond‐swapping and perpetually connected frameworks. For *λ* significantly greater than 1, the system simulates an enduring bond lifespan. Conversely, at *λ* equals 1, the supplementary potential energy accrued from double bond creation is counterbalanced by the magnitude of the three‐body potential. Ciarella and co‐workers further employed this three‐body potential CGMD in conjunction with mode‐coupling theory to systematically uncover the structural basis of the complex glassy dynamics observed in vitrimers.^[^ ^82^ ^]^

(1) $$V_{\mathrm{threebody}\;}=\;\lambda \sum_{ijk}\varepsilon V_{3}\left(r_{ij}\right)V_{3}\left(r_{ik}\right)$$

(2) $$V_{3}\left(r\right)\;=\left\{\begin{array}{cc} 1, & r\leq r_{\min},\\ \;\frac{-V_{\mathrm{bond}\;}\left(r\right)}{\varepsilon }, & \;r_{\min}\leq r\leq r_{\mathrm{cutoff}\;} \end{array}\right.$$

where *r_ij_* represents the distance between particles *i* and *j* and *λ* is a modifiable parameter.

Recently, several combinations of CGMD and MC simulations have been utilized to investigate the effect of bond exchange on the dynamics of vitrimers. These studies could be traced back to the work of Pant and Theodorou^[^ ^84^ ^]^ in introducing an end‐bridging MC move mechanism, which is a generalized version of the concerted rotation algorithms. After the work of Pant and Theodorou, similar algorithms that combine MD and MC simulations have been implemented to investigate materials with reversible bonds, including thermoreversible gels, supramolecular polymers, and telechelic polymers, among others.^[^ ^84^, ^85^ ^]^ For instance, Perego et al.^[^ ^21b^ ^]^ introduced a hybrid MD–MC simulation methodology designed to illuminate the bond exchange mechanisms inherent in vitrimers. Subsequently, this technique was employed to analyze the dynamics and rheology of a representative vitrimer and juxtapose its attributes with those of a permanently crosslinked counterpart. The same research group further leveraged this methodology to probe the microscopic dynamics and the overarching rheology of vitrimers exhibiting rapid bond exchange rates.^[^ ^21d^ ^]^ In addition, they discerned the correlations and distinctions between dissociative and associative networks in their viscoelastic behavior.^[^ ^86^ ^]^

In the MD–MC paradigm, considering a system encompassing several polymer chains, two such chains became covalently linked at time *t = t*_0_. At designated time intervals set by the user, every reactive monomer underwent an examination to discern a suitable candidate for bond exchange. Given that (*i, j*) and (*k, l*) represent pairs of bonds amenable to exchange, and upon identifying a monomer at a distance *r* predefined by the user, the alteration in energy, symbolized as Δ*U*_Exchange_, could be ascertained (see Equation (3)). Should Δ*U*_Exchange_ ≤ 0, the emergent configuration was endorsed. Conversely, if Δ*U*_Exchang_ *_e_* > 0, the modification was sanctioned according to the Boltzmann acceptance criterion:

(3) $$\begin{array}{c} \Delta \;{\tilde{U}}_{\mathrm{Exchange}\;}={\tilde{U}}_{\mathrm{New}\;}\;\left(i,l\right)+{\tilde{U}}_{\mathrm{New}\;}\left(j,k\right)-{\tilde{U}}_{\mathrm{Old}\;}\left(i,j\right)-{\tilde{U}}_{\mathrm{Old}\;}\left(k,l\right) \end{array}$$

In conjunction with the design of BER to simulate exchange reactions, a pivotal consideration in the investigation of vitrimers systems is the construction of the initial system. At the onset of simulations, given an isotropic system, molecules are typically dispersed homogeneously with random configurations, employing 3D periodic boundary conditions (Figure 3d). Conversely, when probing surface properties of vitrimers such as surface welding, the initial configuration of the MD simulation commonly employs periodic boundary conditions in only 2D, allowing for a free surface (Figure 3e). An important component in vitrimer simulations is the establishment of a cross‐linking structure. The MD process of building crosslinking structures has been investigated by many researchers.^[^ ^76^, ^83^ ^]^ This process is generally bifurcated: initially, monomers are interconnected based on a specified set of criteria; subsequently, relaxation occurs, mitigating the artificial energy introduced during the connection phase (Figure 3c). To circumvent excessive artificial energy accumulation during connection, an iterative methodology is commonly adopted, thereby restricting the volume of connection reactions in a single iteration. Another technique often employed is the annealing process and its variants. Typically, the system undergoes a thermocycling process—both heating and cooling—to form the linking network and stabilize the system. For instance, Perego et al.^[^ ^21i^ ^]^ fabricated a stable crosslinked network by reacting the terminal segments of polymer chains with the four reactive segments of crosslinkers via the simulated annealing polymerization method. Once these reactive sites were bonded, the bond lengths of the newly formed connections were methodically shortened to an average magnitude.

### 3.2 Versatile Vitrimers Properties

Two Transition Temperatures: Vitrimer thermodynamics and kinetics are delineated by two pivotal transition temperatures: i) the glass transition temperature (*T*_g_), which is a characteristic feature of fragile glass‐former liquids and ii) the topological freezing temperature (*T*_v_), which is specific to vitrimers and corresponds to changes in the network topology at higher temperatures.^[^ ^5a^ ^]^ Notably, *T*_v_ typically manifests at temperatures surpassing *T*_g_. At these temperatures, the temporal scale of topological alterations protracts upon cooling, leading to decelerated network reshuffling. Empirically, this shift is pinpointed when shear viscosity achieves 10^12^ Pa·s.^[^ ^2h^ ^]^ Several determinants, encompassing monomer and crosslinker chemistry, crosslinker number density, catalyst concentration, cooling rate, and the prevalence of exchangeable bonds, influence the viscoelastic attributes of vitrimers. The optimal design for these materials would ensure they emulate a thermoset network under practical application conditions yet exhibit the malleability of a viscous liquid during elevated temperature processing without compromising network cohesion. Although some have posited the potential of ascertaining *T*_v_ through volumetric analyses akin to the glass transition, current instrumentational constraints render the direct experimental identification of *T*_v_ a formidable endeavor.

Arrhenius Rheological Properties: The zero‐shear viscosity of vitrimers follows an Arrhenius‐like temperature dependence, especially above *T*_v_. This means that as the temperature increases, the viscosity decreases exponentially, governed by the Arrhenius equation. This behavior is closely related to the rate of bond exchange reactions, which increases with temperature.

Stress Relaxation Behavior: Stress relaxation in vitrimers is intrinsically tied to the bond exchange reactions. When a vitrimer is deformed, it can relax the applied stress by undergoing bond exchange reactions that allow it to rearrange its network topology. This process is more pronounced at temperatures above *T*_v_, where these exchange reactions are more frequent. This behavior has significant implications for applications that require materials to hold their shape under continuous mechanical stress. While the stress relaxation capability of vitrimers can be advantageous for certain applications such as self‐healing materials, it can also be a drawback in scenarios where the material's structural integrity must remain unchanged over extended periods.^[^ ^87^ ^]^

Self‐Healing/Surface Welding Characteristics: The bond exchange reactions that grant vitrimers their unique properties also enable self‐healing/surface welding capabilities. When a vitrimer is damaged, it can heal itself by undergoing bond exchange reactions at the site of the damage, re‐forming the network. Two vitrimers pieces can be rejoined without the introduction of additional monomers or chemicals at the interface. This ability is more effective at temperatures above *T*_v_ where bond exchange reactions are more frequent.

The bond exchange reactions are central to the behavior of vitrimers. They dictate the *T*_v_ and are responsible for stress relaxation, Arrhenius rheological behavior, and self‐healing characteristics. As temperature increases, the rate of these reactions increases, leading to decreased viscosity and increased ability to relax stresses and heal damage.^[^ ^88^ ^]^ The *T*_g_ and *T*_v_ serve as critical benchmarks to understand and predict how a vitrimer will behave in a given condition, bridging its macroscopic properties (rheology and self‐healing) with its microscopic bond dynamics. As a result, understanding bond exchange reactions is a key step to controlling transition temperatures as well as the macroscopic properties of vitrimers.

**Figure 4:**
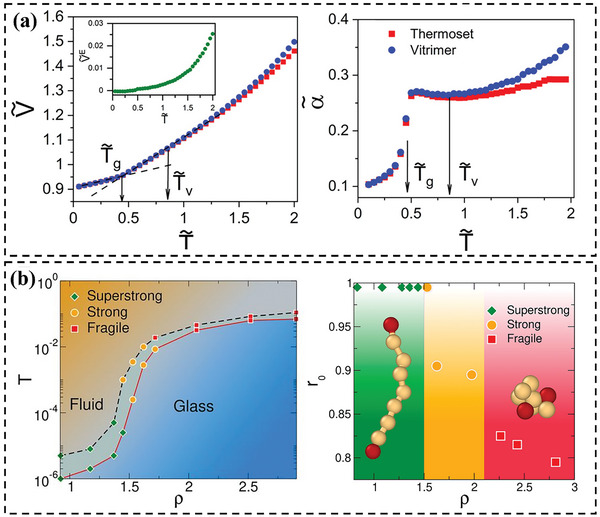
a) Reduced (left) specific volume and (right) thermal expansion coefficient as a function of reduced temperature *T̃* (*T̃* = *K*_B_ *T*/*ε*, *k*_B_ is the Boltzmann constant, *ε* is the well depth of Lennard–Jones potential), *T̃*_g_ and *T̃*_v_ denote the glass transition temperature and the topology freezing temperature, respectively. Reproduced with permission.^[^ ^21b^ ^]^ Copyright 2020, American Chemical Society. b) Simulated phase diagram as a function of temperature *T* and bulk density (left). Distance *r*_0_ corresponds to the main peak of the radial distribution function g(r) as a function of vitrimer bulk density (right). Reproduced with permission.^[^ ^82^ ^]^ Copyright 2019, PNAS.

Simulation methods offer a robust approach to understanding the intricate behaviors of vitrimers. These simulations provide insights at the molecular level, allowing researchers to probe and predict macroscopic behaviors. For example, Perego and Khabaz^[^ ^21b^ ^]^ proposed a hybrid MD–MC simulation approach aimed at elucidating bond exchange processes in vitrimers. Experiments have shown that vitrimers manifest a terminal regime in the elastic modulus as a function of frequency, attributable to augmented mobility and topological alterations. In contrast, thermosets exhibit an equilibrium elastic modulus at lower frequencies.^[^ ^89^ ^]^ Their MD simulation captures the rheological behavior of vitrimers within the terminal regime, corroborating experimental observations. Remarkably, the model effectively anticipates two transition temperatures (*T*_g_, *T*_v_) for vitrimers. *T*_g_ is inferred from the volumetric data and *T*_v_ is determined by the thermal expansion coefficient *α̃* at different temperatures (**Figure** **4**a). In the glassy state, vitrimers and thermosets exhibit equivalent mobility. Elevated temperatures induce diffusive tendencies in vitrimers. Rheological analyses highlight vitrimers' characteristic terminal elastic modulus at minimal frequencies and their zero‐shear viscosity's Arrhenius‐like temperature dependence at temperatures above *T*_v_. Simulations suggest bond lifetimes critically shape network dynamics. Deformation velocity relative to bond exchange rate dictates thermoset or viscous behavior. Ciarella et al. examined star polymer vitrimers and elucidated the associations between microstructure and fragility.^[^ ^82^ ^]^ The MD simulations demonstrate that by modulating the bulk density, the fragility of vitrimers at *T*_g_ can be adjusted, transitioning from fragile (super‐Arrhenius) to robust (Arrhenius) and even to exceptionally resilient (sub‐Arrhenius) behavior (Figure 4b). Notably, such fragility and its density‐driven adaptability appear to be decoupled from the *T*_v_, beyond which all bond‐exchange phenomena desist.

**Figure 5:**
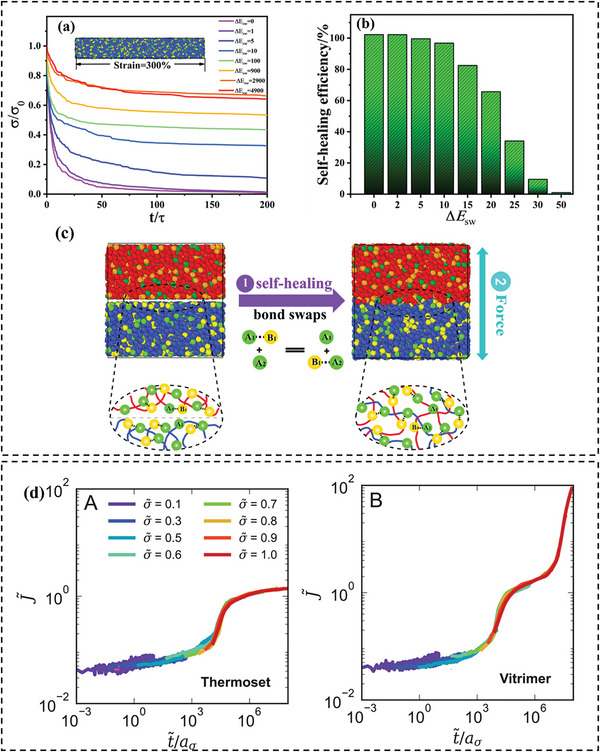
a) Normalized stress relaxation under the constant uniaxial strain of 300% imposed as a step at *t* = 0 for a range of bond swap energy barriers. b) Self‐healing efficiency for different bond swap energy barriers. c) Schematic of self‐healing. Reproduced with permission.^[^ ^21c^ ^]^ Copyright 2022, American Chemical Society. d) Universal curves of the creep compliance as a function of reduced time for (left) thermoset and (right) vitrimer. Reproduced with permission.^[^ ^21i^ ^]^ Copyright 2022, Wiley.

The bond exchange mechanism endows vitrimers with the capability to reconfigure and modulate their structure temporally, even amidst sustained strain, culminating in discernible stress relaxation. MD simulations proffer intricate insights at the microscopic level, facilitating the exploration of atomic and molecular interactions pivotal to the stress relaxation phenomena observed in vitrimers. For instance, Zhao et al.^[^ ^21c^ ^]^ utilized CGMD simulations to develop a representative vitrimer system comprising a polymer network constructed from linear chains, capable of reconfiguring the network topology through BERs. The present study investigates the impact of bond swap energy barrier (Δ*E*_sw_) and temperature on the kinetics of BERs, uniaxial stress–strain response, stress relaxation, self‐healing efficacy, and extrusion reprocessing of a bulk vitrimer. MD simulations show that with a rise in temperature, the polymer chains exhibit pronounced mobility and diffusive characteristics.^[^ ^21c^ ^]^ Such behavior aligns with experimental observations of the viscoelastic liquid dynamics of vitrimers that allow them to be malleable at high temperatures.^[^ ^90^ ^]^ Stress relaxations were investigated for various Δ*E*_sw_ systems (**Figure** **5**a). As Δ*E*_sw_ diminished, the BER was activated, leading to an accelerated rate of stress relaxation. By performing a triaxial deformation to induce the cavities, the vitrimer was able to demonstrate its outstanding self‐healing ability by reducing Δ*E*_sw_, as well as increasing the self‐healing time and temperature (Figure 5b,c). This trend can be attributed to the fact that bond exchanges at the interface foster surface amalgamation. A diminished potential barrier accelerates interface fusion, thereby augmenting self‐healing efficiency. In addition, in a recent study conducted by Ciarella and colleagues,^[^ ^73d^ ^]^ the adhesion behavior of two star shape vitrimer samples was examined using CGMD simulations. The results revealed that the samples bonded together on timescales significantly shorter than the stress relaxation time through the swap mechanism. The study further demonstrated that the swap mechanism facilitated the diffusion of the star shape vitrimers through the material via coordinated swap events. However, the healing process was observed to occur much more rapidly and was not found to be dependent on this mobility. It is because the healing depends on the mobility of the arms but not the mobility of the whole star polymer.

The ability to exchange covalent bonds of vitrimers can be harnessed to enable self‐healable aerospace composites where damage can be reversed through heating or recyclable matrix materials where the matrix can be reclaimed after use.^[^ ^2^, ^91^ ^]^ Singh et al.^[^ ^78^ ^]^ simulated vitrimer and analyzed their thermomechanical response using MD simulation. The simulation of healing a cylindrical pore caused by carbon nanotube pullout demonstrated the model's ability to showcase both vitrimer healing and the full recovery of elastic modulus upon cooling. In addition, Park et al.^[^ ^73a^ ^]^ investigated the self‐healing behavior of a vitrimer nanocomposite system, which involved cut, BER loop (healing), and tension. The simulation results indicated that significant self‐healing occurred above the *T*_g_ of the material, which was consistent with experimental observations of various vitrimers. Moreover, the vitrimer nanocomposite demonstrated superior self‐healing properties compared to the vitrimer model at all three temperatures investigated. This enhanced self‐healing capability was attributed to the decrease in *T*_g_.

As a result of the self‐healing mechanism, vitrimers also exhibit welding capabilities. Most of the earlier MD studies investigating adhesion and welding at polymer interfaces employ a bead‐spring model^[^ ^92^ ^]^ that captures the properties of linear homopolymers.^[^ ^93^ ^]^ For example, Pierce et al.^[^ ^94^ ^]^ conducted a study to explore the dynamics of polymer chains at an interface, revealing that the motion of chain ends has a substantial impact on interdiffusion across specific interfaces. Ge et al.^[^ ^95^ ^]^ has examined the interfacial strength of polymers and demonstrated that it is correlated with the formation of entanglements at the interface during welding. Recently, Bukowski et al.^[^ ^96^ ^]^ combined experiment and CG bead‐spring simulations to investigate the impact of entanglements on the deformation mechanisms of thin glassy polymer blend films. This method enabled the examination of both macroscopic and molecular perspectives to obtain a multi‐length scale comprehension of polymer strength. Despite that, the application of full atomistic MD simulations to investigate the network rearrangement of vitrimers has only been effectively implemented in recent years.

Yang et al.^[^ ^76^, ^83^ ^]^ used atomistic MD simulation to explore surface welding, which resulted from bond exchange reactions in a vitrimer system (diglycidyl ether of bisphenol A as the monomer and tricarballylic acid as the crosslinking agent).^[^ ^83^ ^]^ The simulations showed how the bond exchange reactions happen and the path of active atoms at the interface during surface welding. The study then investigated the effect of welding conditions on the mechanical properties of the welded materials. The welding conditions examined included welding time, welding temperature, degree of polymerization, and crosslinking density of the original networks. It was found that the modulus and yield strength of welded materials increased with the duration and temperature of welding, and eventually approached the values of the fresh sample. In addition, a decrease in the crosslinking density resulted in a shorter welding time. Notably, the study also found that a decrease in the degree of polymerization led to slower surface welding between covalent adaptable networks. The penetration depth of polymer chains across the interface suggested that the entanglement of polymer chains significantly reduced the diffusivity. Following the success of those previous studies, the authors applied the MD method to investigate the surface welding and shape memory behaviors of Diels–Alder (DA) network (vitrimer‐like materials).^[^ ^97^ ^]^ The uniaxial tension measurements were utilized to investigate the mechanical characteristics of the fresh and fully welded networks. The outcomes demonstrated that the mechanical properties of the welded networks were completely regained by enough welding time, which was analogous to those of the fresh network. The results revealed that the modulus and maximum stress were increased with welding time due to the increased DA reactions. Ultimately, they attained the identical value as the fresh samples. While this study successfully demonstrated the evolution of mechanical properties and shape memory behavior of a covalent adaptable (DA) network during welding, the depolymerization process resulting from retro‐DA reactions at high temperatures was not investigated. As the degree of depolymerization was significantly influenced by temperature, it is important to investigate the effect of temperature on the surface welding behavior of DA networks. The authors further improved the study by proposing an MD simulation methodology that incorporated the retro‐DA and DA reaction sequences to examine the surface welding behavior of thermally reversible DA networks.^[^ ^76c^ ^]^ Taking advantage of the van't Hoff Equation, they elucidated the correlation between temperature and equilibrium conversion for the studied temperature‐dependent reversible network. Similarly, Zhang et al.^[^ ^98^ ^]^ used the same technique to show that BERs could improve the diffusion rate and the final penetration depth in crosslinked epoxy‐polyimine networks.

Despite their numerous beneficial attributes, vitrimers also exhibit certain significant limitations. Specifically, vitrimers are known to experience substantially more creep than commercially available thermosets, which has been reported in numerous experiments.^[^ ^7^, ^89^, ^99^ ^]^ As creep refers to the proclivity of a material to undergo continuous and permanent deformation under stress, this characteristic poses a considerable disadvantage for potential vitrimer end products. Creep is highly undesirable in the majority of applications where thermosets or elastomers are customarily employed.^[^ ^5^, ^100^ ^]^ In this regard, Perego et al.^[^ ^21i^ ^]^ conducted CGMD‐MC simulations to investigate the creep and recovery behavior of thermoset and vitrimer. Their study showed good agreement with reported experimental data for temperature‐dependent creep compliance. The simulations demonstrated that once the vitrimer reached equilibrium creep compliance, creep was no longer suppressed, and the material continued to deform under shear load, indicating stress‐relaxation events occurring within the network (Figure 5d). In a recent study, Singh et al.^[^ ^101^ ^]^ utilized MD simulations to investigate the creep behavior of a vitrimer system comprised of diglycidyl ether of bisphenol‐A cross‐linked with 4‐aminophenyl disulfide. The investigation revealed that vitrimers experienced a reduction in their available free volume due to the dynamic rearrangement of bonds under tensile loads. They found that there was a distinct preference for alignment of dynamic bonds perpendicular to the loading axis in vitrimers. This preference led to a decrease in axial stiffness during secondary creep; and consequently, resulted in greater creep strain compared to epoxies. This increased strain over extended time periods contributed to the growth of voids, ultimately leading to tertiary creep.

Swelling is another phenomenon that can significantly affect the performance of vitrimers, and its behavior can vary greatly depending on the specific vitrimer system. Smallenburg et al.^[^ ^31^ ^]^ investigated the behavior of vitrimers in the presence of a solvent using a patchy particle model, which allowed them to make predictions about the changes in network structure and topology during swelling. It should be noted that the model does not explicitly consider solvents. By lowering the density in the model system, they were able to construct a phase diagram representing the behavior of vitrimers in a good solvent. Notably, their model predicted that when in contact with a solvent reservoir, the vitrimer network did not dissolve but instead underwent particle expulsion and composition changes, ultimately approaching a defect‐free state. Solvent could strongly affect the mechanical properties of vitrimers. Sun et al.^[^ ^102^ ^]^ conducted a study to investigate the effect of solvent evaporation on the polymerization of vitrimers with different solvents, including ethylene glycol (EG) and octamethylene glycol (OG). The study demonstrated that the evaporation in the polymer‐EG system could lead to a more uniform distribution of chain segment lengths in the network as fewer solvent molecules were trapped in the solution film. Their research further revealed that the size of the solvent molecules had a negligible effect on the evaporation rate during the early stages before the formation of the polymer‐rich layer. However, after this stage, the polymer‐OG system, which contained larger solvent molecules, exhibited slower evaporation and polymerization. On the opposite side, introducing solvent would lead to the depolymerization of the system. In addition, the same group investigated the decomposition of an epoxy vitrimer in EG solvent via transesterification‐type BER.^[^ ^103^ ^]^ Their findings revealed that vitrimer decomposition in EG solvent was governed by the diffusion of polymer chain segments into the solvent. As the decomposition progressed, chain segments amassed at the polymer–solvent interface, culminating in the formation of a dense gel layer. Their research informed the suitable choice of processing conditions for the effective recycling of vitrimers.

### 3.3 Computational Aids in Designing Vitrimers With Targeted Properties

The mechanical and rheological properties of vitrimers hold a crucial position in establishing their applicability across a diverse array of industries. These properties not only dictate the material's performance characteristics but also influence its processability, thereby making them important to consider in the development of vitrimers for specific applications. A key factor in controlling these properties lies in modulating the rate of BERs within the vitrimer network. By judiciously adjusting the BERs, it becomes possible to achieve plastic processing and recycling; while, maintaining compatibility with existing processing technologies.

Computational simulations, such as MD and MC methods, have emerged as valuable tools for elucidating the interplay between BERs and vitrimer properties. These techniques can provide critical insights into the underlying structure–property relationships, guiding the rational design of vitrimers with targeted characteristics. Further, DFT calculations can offer valuable information on the mechanisms governing BERs, enabling the fine‐tuning of reaction rates and, in turn, the optimization of vitrimer properties. Consequently, the synergistic combination of computational approaches paves the way for the development of novel vitrimer materials with tailored mechanical and rheological properties, catering to the diverse needs of various applications.

#### 3.3.1 Tuning Bond Exchange Reaction

**Figure 6:**
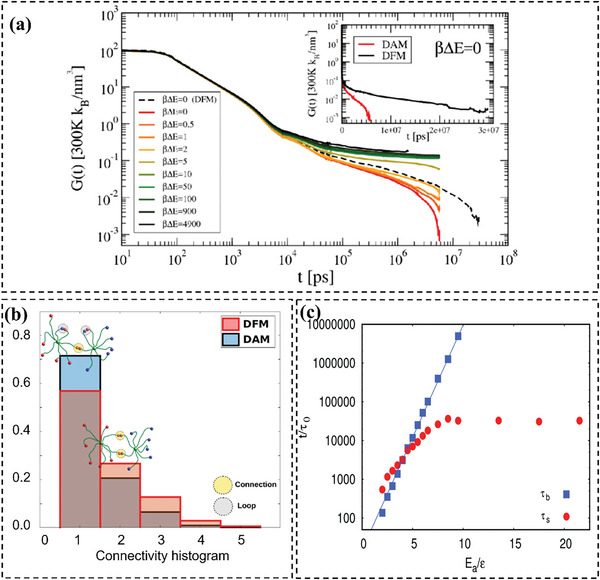
a) Comparison between the defect allowing mixture (DAM) and defect free mixture (DFM) stress relaxation values. b) Histogram of the number of connections between connected stars for both mixtures. Reproduced with permission.^[^ ^45^ ^]^ Copyright 2018, American Physical Society. c) Dependence of the bond lifetime *τ*_b_ and the slow relaxation time *τ*_s_ for several *E*_a_ values. Reproduced with permission.^[^ ^53^ ^]^ Copyright 2019, Royal Society of Chemistry.

As previously mentioned, MD simulations are instrumental in providing insights into the methods by which materials can be tailored to achieve the desired mechanical properties. These computational approaches enable a deeper understanding of the relationships between material structures and their properties, facilitating informed decision‐making in material design and optimization. For example, Simone Ciarella and co‐workers^[^ ^45^ ^]^ proposed a CG model to investigate stress relaxation in star‐polymer networks induced by dynamic bond‐exchange processes. Utilizing MD simulations, the researchers determined the stress relaxation modulus, revealing a transition from solid‐like to liquid‐like behavior as the bond‐exchange rate increased. In addition, the study highlights a remarkable difference in stress relaxation stemming from molecular topology, demonstrating that networks constructed from building blocks permitting loop formation through bond exchange exhibit significantly faster stress relaxation than those comprised of loop‐preventing building blocks, even when bond‐exchange rates are identical (**Figure** **6**a). Topological defects, in the form of loops, are found to serve as highways to stress relaxation, giving faster self‐healing, malleability, and recyclability. The presence of loops reduces the average number of connections between star polymers, thereby decreasing redundancy (Figure 6b). Consequently, this increases the likelihood that a single swap event will disconnect two star‐polymers, which in turn accelerates stress relaxation. The findings indicate that innovative vitrimer systems can be deliberately designed to incorporate defects as a strategy for modulating their mechanical properties.

Another example of MD‐aided vitrimer design was reported by Wu. et al.^[^ ^53^ ^]^ Experiment shows that the vitrimer displays a double relaxation behavior, characterized by a rubbery plateau at elevated frequencies and a subsequent plateau at diminished frequencies, culminating in terminal relaxation.^[^ ^104^ ^]^ Their MD simulations further corroborate this finding, revealing dual plateaus in the intermediate scattering curves; thus, substantiating the observed double relaxation behavior. The simulation results reveal that BERs affect the diffusion mode of the constituent molecules, thereby influencing the BER and other relaxation dynamics (Figure 6c). Using the BER model in conjunction with a CG model, the properties of specific vitrimers can be reproduced by altering relevant parameters such as intermolecular interaction, bond angle, and bond length. The discoveries offer insight into the molecular mechanism of vitrimers’ dynamic behavior and present a method for their rational design, suitable for existing processing techniques such as injection molding and extrusion molding.

**Figure 7:**
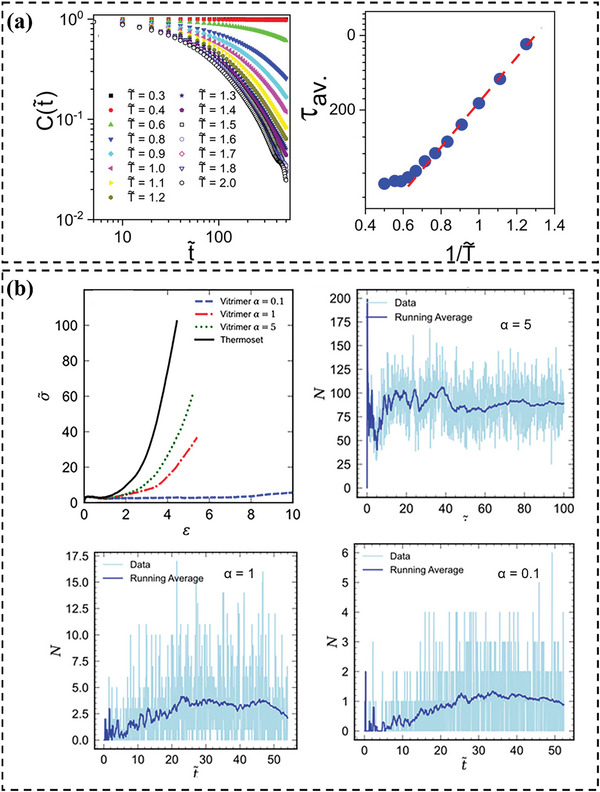
a) Autocorrelation function of exchangeable bonds as a function of simulation time at different temperatures (left). Average lifetime of bonds as a function of temperature (right). Reproduced with permission.^[^ ^21b^ ^]^ Copyright 2020, American Chemical Society. b) Stress–strain relations of the simulated thermoset and vitrimer system for various values of *α*. Number of successful bond exchanges as a function of reduced time during uniaxial extension simulations of vitrimers for various *α* values (*α* is an artificial parameter, which is used to scale the energy difference between two states in the MC step). Reproduced with permission.^[^ ^21g^ ^]^ Copyright 2021, Wiley.

Alessandro Perego and Fardin Khabaz^[^ ^21b^ ^]^ used the CGMD‐MC method to investigate the network integrity and flowability of vitrimers. The study indicated that the lifetime of exchangeable bonds plays a crucial role in determining the rheology and dynamics of vitrimer networks (**Figure** **7**a). At moderate to high temperatures (*T̃* ≤ 1.7), the average lifetime of exchangeable bonds exhibits an Arrhenius‐like temperature dependence. However, at exceedingly high temperatures (*T̃* ≥ 1.8), the bond lifetime becomes unresponsive to temperature variations. Moreover, factors such as network architecture and interaction parameters can significantly impact their rheological properties and thermodynamics. The current model can be used to optimize the rheological and mechanical properties of vitrimers for processing and end‐use applications. Following this, the authors further developed a similar but more elaborate strategy. They employed a hybrid MD–MC technique to scrutinize the correlation between the energy barrier of bond exchange reactions and the dynamics and mechanical properties of vitrimers.^[^ ^21g^ ^]^ The dynamic and mechanical properties of the vitrimer system are profoundly affected by the number of successful bond exchanges happening at every step (Figure 7b). Lowering the energy barrier of the bond exchange reaction enhances the mobility of vitrimer segments in both rubbery and glassy regimes, influencing the strain–stress relationship of these networks. A higher number of exchanges results in more significant deformation before fracture, even at low temperatures. The results presented provide valuable insight to accelerate the inverse design of vitrimers that can replace conventional thermosets, optimizing their rheological and mechanical properties critical for processing and end‐use applications.

Experimental studies often provide valuable insights into the macroscopic behavior of vitrimers, such as their mechanical, rheological, and thermal properties. However, they may not fully elucidate the microscopic processes responsible for these properties. By examining the reaction mechanisms, researchers can establish a direct link between the molecular‐level phenomena and the observed macroscopic behavior. DFT is the method of choice in studying the reaction mechanism due to its ability to accurately identify the underlying BERs, transition states, intermediates, and activation barriers that govern the dynamic nature of vitrimers. Currently, most of the DFT calculations are used to assist experimental studies in the explanation of the underlying mechanism.

**Figure 8:**
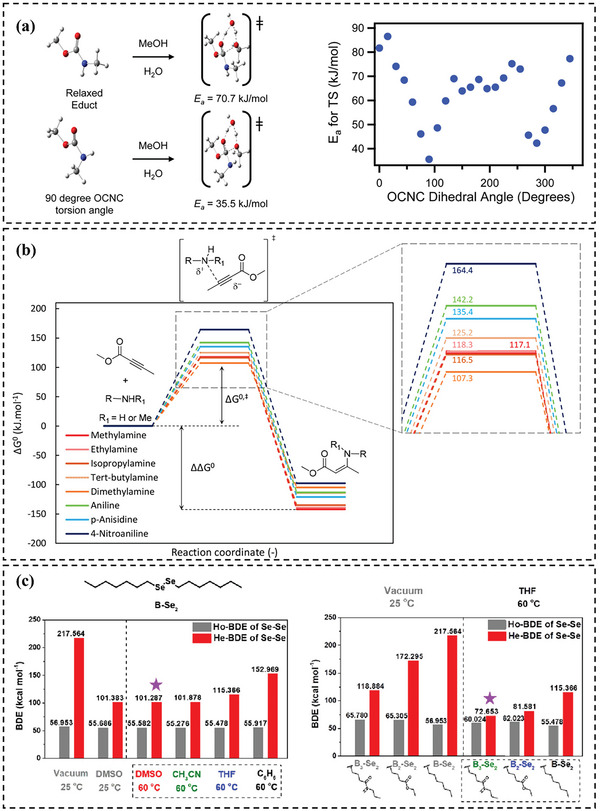
a) The structures of *N,O*‐dimethylcarbamate and activation energy *E*_a_ as a function of the O═C(OCH_3_)N(H)CH_3_ dihedral angle. Reproduced with permission.^[^ ^20i^ ^]^ Copyright 2015, American Chemical Society. b) Calculated free energy diagram for the reaction between methyl but‐2‐ynoate with different amines. Reproduced with permission.^[^ ^7c^ ^]^ Copyright 2021, American Chemical Society. c) Bond dissociation energy (Ho‐BDE) and heterolytic bond dissociation energies (He‐BDE) of the Se─Se bond in Bn‐Se_2_ with increasing solvent polarity and the polarity of the molecular groups. Reproduced with permission.^[^ ^105^ ^]^ Copyright 2022, American Chemical Society.

For example, Fortman et al.^[^ ^20i^ ^]^ introduced a new category of vitrimers, namely, polyhydroxy urethanes (PHUs), which could be reprocessed without the aid of an external catalyst at high temperatures and pressure. The authors hypothesized that transcarbamoylation reactions may be mechanically activated because of the variation in activation energy between PHU stress relaxation and model compound transcarbamoylation and the inoperative dissociative exchange processes. To test this hypothesis, the researchers performed DFT calculations to predict the activation energy values (*E*_a_) for the water‐catalyzed reaction of *N,O*‐dimethylcarbamate with methanol to form the product orthocarbamate. To investigate the influence of torsional strain, DFT calculations were employed to determine the energies of reactant and transition‐state with different fixed OCNC dihedral angles of O═C(OCH_3_)N(H)CH_3_ (**Figure** **8**a). Notably, it was found that the activation energies associated with many torsionally strained educt and TS structures were significantly lower than those for the equivalent fully relaxed reaction. Specifically, when the OCNC torsion angle was 90°, the predicted activation energy dropped to 35.5 kJ mol^−1^, in contrast to the activation energy of 70.7 kJ mol^−1^ predicted for fully relaxed structures in this model reaction (Figure 8a). The observed decrease in activation energy due to torsional strain supported the hypothesis that transcarbamoylation reactions were mechanically activated.

In a recent study, Spiesschaert et al.^[^ ^7c^ ^]^ has demonstrated the feasibility of utilizing alkyne esters (AE) as a substitute building material for the synthesis of vinylogous urethane (VU) vitrimers through polyaddition polymerization with amines. This method leads to water‐free formulations and enables easy access to high‐quality cured VU vitrimer materials, without any defects. In their study, DFT calculations were also conducted to provide a rationale for the experimental findings on the formation of VUs in terms of steric and electronic influences and to investigate their impact on the transamination reaction. To simplify the calculations, the study focused on simplified versions of both the amines and the acetoacetates (AA) or AE by using methylamine, methyl acetoacetate (Me‐AA), and methyl but‐2‐ynoate (Me‐AE). Gibbs free energy profiles were procured for both the VU formation reaction (originating from AA and AE) and the transamination reaction between an amine and a VU. This led to the determination of the relative Gibbs free energy (ΔΔ*G* ^0^) and the relative Gibbs free energy of the transition state (Δ*G* ^0,‡^) for the rate‐limiting step of the pathway (Figure 8b). To examine the steric and electronic effects, a diverse array of amines, spanning from methylamine to 4‐nitroaniline, was selected. The study discovered that the reactions between amines and Me‐AE were significantly exergonic (ΔΔ*G* ^0^ < −95 kJ mol^−1^), whereas reactions with Me‐AA were predominantly exergonic (−30 kJ mol^−1^ < ΔΔ*G* ^0^ <15 kJ mol^−1^), thereby possessing a diminished thermodynamic driving force. Chen et al.^[^ ^105^ ^]^ also utilized DFT calculations to demonstrate the significant impact of polarity on the heterolytic bond dissociation energies of diselenide exchange reactions. The authors have calculated the homolytic bond dissociation energy (Ho‐BDE) and heterolytic bond dissociation energy (He‐BDE) of the Se─Se bond under different conditions (Figure 8c). They found that the dissociation energy of Se─Se bond decreased remarkably as solvent polarity and the molecular groups’ polarity increased; while, that of the Se─Se bond remained almost unchanged. These outcomes of the theoretical study were in agreement with experimental observations.

Quienne et al.^[^ ^106^ ^]^ employed DFT calculations to demonstrate the crucial role played by the *β*‐OH substituent in facilitating proton transfer by H‐bonding assistance relative to the alkylamine. This *β*‐OH substituent was capable of participating in both intra‐ and intermolecular proton transfer chains; thus, significantly reducing the activation barrier. Berne et al.^[^ ^20a^ ^]^ conducted a related study investigating the electronic influence of the *α*‐CF_3_ substituent, which contributed to the acceleration of the transesterification process. The calculations indicated that while the electron‐withdrawing effect of the *α*‐CF_3_ group played a role in reducing the transesterification activation barrier, its accelerating influence was likely not as significant as the presence of a *β*‐OH substituent on the departing alcohol moiety. In addition, Luzuriaga et al.^[^ ^107^ ^]^ explored the mechanochromic effects evident in epoxy networks featuring 4‐aminophenyl disulfide. Utilizing DFT calculations, the distinctive behaviors between these networks were attributed to a pronounced dipole moment variation when sulfur and nitrogen atoms occupied para positions. The electron transfer dynamics from the donor amino group to the sulfur atom were elucidated through DFT, corroborated by Mulliken charge and spin density values.

#### 3.3.2 Entropy Driven Approach

Recently, a novel form of vitrimer, which utilizes linkers for mediation, has been developed by employing dioxaborolanes in a metathesis reaction. This reaction allows for the formation of cross‐links between polymers and the dynamic modification of the polymer network.^[^ ^14b^ ^]^ The vitrimers exhibit excellent resistance to mechanical, thermal, and chemical factors and are processable like thermoplastics,^[^ ^108^ ^]^ while the underlying design principles remain unclear.

**Figure 9:**
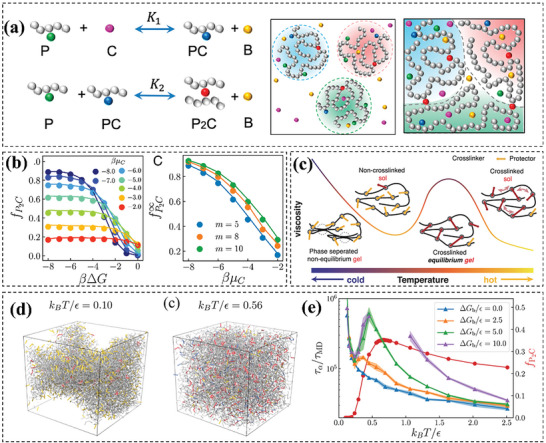
a) Illustration of the two‐step metathesis reactions, the heterogeneous dilute and homogeneous dense systems of the vitrimers. b) Entropy‐driven cross‐linking at low temperature. Reproduced with permission.^[^ ^24^ ^]^ Copyright 2020, PNAS. c) Schematic diagram of the entropy‐driven crosslinking induced sol–gel transition to form thermo‐gelling vitrimer. d) Snapshots of structural change at different temperature. e) Thermo‐gelling vitrimers, structural relaxation time, and crosslinking degree as a function of temperatures for various activation barriers. Reproduced with permission.^[^ ^21a^ ^]^ Copyright 2022, American Chemical Society.

Ran Ni and co‐workers^[^ ^24^ ^]^ proposed a theoretical framework for elucidating the phase behavior of linker‐mediated vitrimers (**Figure** **9**a), with a focus on the role played by entropy. Using mean field theory and CG computer simulations, the authors demonstrated that the entropy of free linkers plays a crucial and non‐trivial role in the behavior of the system. Specifically, as the concentration of free linkers increases, the vitrimer system undergoes a reentrant gel‐sol transition. Intriguingly, even at low temperatures (*β*∆*G* → −∞) or in the dilute limit of byproduct molecules (*βµ*_B_ → −∞), the system is not fully cross‐linked. However, the cross‐linking degree can still be adjusted by altering the concentration of free linkers or the precursor density on polymer chains due to the competition between the conformational entropy of polymers and the translational entropy of linkers. Simulations demonstrate that both cross‐linking degree of system ($f_{P_{2}C})$ increase and plateau at low *β*∆*G*. At low temperature limits, $f_{P_{2}C}^{\infty }$ decreases with increasing *βµ*_C_, which is in line with theoretical predictions (Figure 9b). The mean field theory is in excellent agreement with the results of the CG computer simulations and provides valuable insights for the rational design of linker‐mediated vitrimers with desired properties.

Thereafter, Ran Ni et al.^[^ ^21a^ ^]^ have expanded on their previous work^[^ ^24^ ^]^ by proposing a novel reversible thermo‐gelling vitrimer (Figure 9c) using previously developed mean‐field theory to explain the entropy‐driven crosslinking mechanism. Unlike thermo‐gelling systems that rely on weak interactions such as hydrogen bonds, the novel vitrimers are always crosslinked at high temperatures due to entropy, which is essentially a thermo‐crosslinking elastomer. The addition of protector molecules prevents crosslinking, keeping the polymer in a liquid state, but an entropy‐driven crosslinking occurs with increasing temperature to induce the sol–gel transition. MD simulations indicate that the activation barrier Δ*G*_b_ of the metathesis reaction plays a crucial role in the thermo‐gelling process, which can be controlled experimentally using different catalysts to drive the vitrimer to form an equilibrium gel at high temperature that is not subject to any thermodynamic instability. Structural relaxation time *τ*_α_ and crosslinking degree $f_{P_{2}C}$ were compared. Δ*G*_b_ = 0, *τ*_α_ decreases monotonically with increasing temperature despite increased crosslinking. With higher Δ*G*_b_, crosslinking effects become more pronounced, and *τ*_α_ develops a peak. The system exhibits gelation at both low and high temperatures with distinct properties: low‐temperature gels undergo kinetically arrested phase separation; while, high‐temperature gels remain in equilibrium as homogeneous gels without structural change (Figure 9d,e). The mechanical properties of vitrimers can be reversibly tuned in situ by altering the activation barrier and/or adjusting the concentration of protector molecules. This innovation opens up possibilities for designing and fabricating novel vitrimers for use in biomedical applications.

#### 3.3.3 Vitrimer Composites

Vitrimer composites integrate the dynamic and recyclable nature of vitrimers with the enhanced mechanical, thermal, and other functional properties provided by incorporated fillers or reinforcements. Computational studies play a crucial role in the development of vitrimer composites by providing insights into the fundamental structure–property relationships, reaction mechanisms, and dynamic behavior of these materials. These studies can help guide the design of new vitrimer composites with enhanced properties and functionalities for various applications.

**Figure 10:**
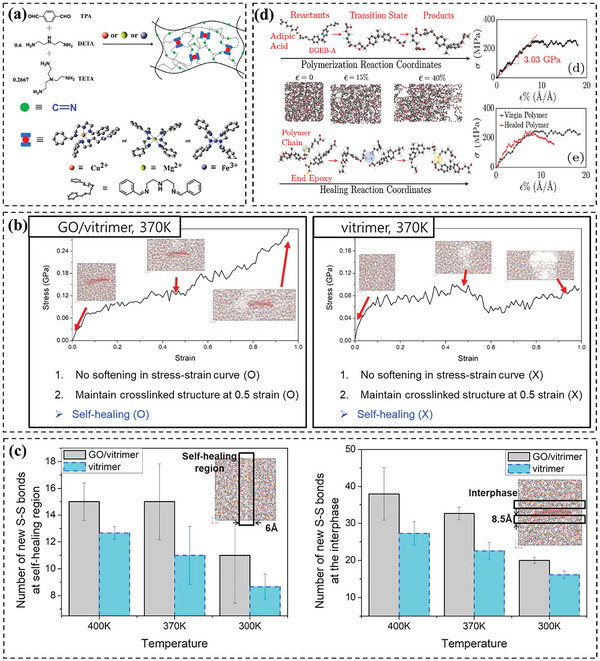
a) Scheme of synthetic route of the polyimine–metal complex vitrimers. Reproduced with permission.^[^ ^111^ ^]^ Copyright 2020, American Chemical Society. b) Self‐healing simulation results of GO/vitrimer and vitrimer, 370 K. c) Percentage of new disulfide bonds as the BER loop count increases and number of new disulfide bonds at the end of the BER loop in the self‐healing region and the interphase. Reproduced with permission.^[^ ^118^ ^]^ Copyright 2020, Elsevier. d) Simulating the vitrimer and its healing using MD simulation. Reproduced with permission.^[^ ^73b^ ^]^ Copyright 2021, Elsevier.

As previously elucidated, one inherent limitation of vitrimers is the phenomenon of creep. To overcome this drawback, one potential strategy is to introduce permanent cross‐links into the vitrimeric network. In a pioneering effort, Zhang and co‐workers.^[^ ^109^ ^]^ introduced Schiff base structure into cross‐linked polymers, resulting in polyimines with exceptional malleability and recyclability. Besides its dynamic nature, the Schiff base functionality also enables its use as a metal complex ligand, playing a crucial role in the advancement of coordination chemistry.^[^ ^110^ ^]^Latest research has focused on enhancing the creep resistance, thermal and mechanical properties, and chemical stability of vitrimers based on the Schiff base. One such strategy proposed by Sheng Wang and co‐workers.^[^ ^111^ ^]^ involves incorporating metal complexes into cross‐linked polyimine vitrimers for the first time. The researchers selected Cu^2+^, Mg^2+^, and Fe^3+^ metal ions and optimized different probable structures of metal complexes using DFT computational calculation. Scheme of synthetic route of the polyimine–metal complex vitrimers is illustrated in **Figure** **10**a. They systematically investigated the effect of Cu^2+^ content and different metal complexes on the properties of polyimine–metal complex vitrimers, including creep resistance, stress relaxation, thermal and mechanical properties, solvent resistance, thermal stability, and recyclability. The findings showed that incorporating metal complexes can significantly reduce the creep of polyimine vitrimers. The additional amount of metal ions can also adjust the vitrimer's initial creep temperature, creep degree, and activation energy. Further, the introduction of coordination structures significantly enhances the thermal and mechanical properties, organic solvent, and acid resistance of the polyimine vitrimer. This enhancement results from the increased cross‐link density and rigidity of the segmental chain. The polyimine–metal complex vitrimers demonstrate excellent reprocessing recyclability. Overall, this elegant approach highlights that incorporating metal coordination into Schiff‐base vitrimers effectively enhances high‐temperature creep resistance, thermal/mechanical properties, and chemical stability.

To improve vitrimers’ mechanical properties, researchers have used nanofillers as reinforcing agents, including graphene oxide (GO), which has been shown to enhance self‐healing properties.^[^ ^112^ ^]^ This was confirmed in studies by Krishnakumar et al.^[^ ^113^ ^]^ In an article by Chanwook Park and co‐workers,^[^ ^73a^ ^]^ MD simulations were used to investigate the filler effect of vitrimer nanocomposites. Their study, the first of its kind, showed that adding GO nanofillers induced self‐healing properties at an atomic level. To assess the self‐healing performance, a self‐healing simulation containing a series of cut‐BER (healing)‐tension was proposed, and the self‐healing properties of GO/vitrimer nanocomposites and pristine vitrimers were compared. The findings showed a significant self‐healing process occurring above the *T*_g_ of the material. The GO/vitrimer models exhibited better self‐healing properties than vitrimer models, which can be attributed to the reduced *T*_g_ of GO/vitrimer (Figure 10b). The authors quantified the number of newly formed disulfide bonds during the BER loop and found a consistent increase in the number of new disulfide bonds during the self‐healing simulation at all different temperatures in GO/vitrimer nanocomposites (Figure 10c). This observation provides compelling evidence that incorporating GO into the vitrimer facilitates the activation of dynamic bond exchange reactions, consequently yielding enhanced self‐healing performance and quality. The reduction in *T*_g_ in polymeric nanocomposites is a general phenomenon observed in various filler/matrix compositions. Therefore, the results of this study can be extended to different nanofillers/vitrimer composites.

Despite the considerable amount of research conducted on vitrimers, their fatigue behavior remains an unexplored area of investigation.^[^ ^2^, ^5^ ^]^ Fatigue‐induced failure is the leading cause of catastrophic failure in carbon‐fiber reinforced polymeric (CFRP) composites, but this has not been reported in prior research. Traditional methods to mitigate fatigue involve the use of nano‐scale additives (such as nano‐silica,^[^ ^114^ ^]^ graphene,^[^ ^115^ ^]^ and carbon nanotubes^[^ ^116^ ^]^) that hinder crack propagation in the polymer, extending fatigue‐life. However, these methods do not prevent eventual failure as crack growth is slowed and not reversed. Alternatively, self‐healing polymers^[^ ^117^ ^]^ that release a curing agent to repair local damage have been studied, but this approach also fails to address fatigue. Once the curing agent is released, it is consumed and cannot be reused. To tackle the irreversibility of fatigue, Mithil Kamble et al.^[^ ^73b^ ^]^ reported a vitrimeric matrix and created vitrimeric CFRP (vCFRP), for which accumulation of fatigue damage in the vitrimer could be “reversed” by periodic healing at temperatures above the topology freezing transition temperature. This approach demonstrates that fatigue‐induced failure in vitrimers and vCFRP can be indefinitely postponed. As shown in Figure 10d, utilizing MD simulations, they investigated the tensile modulus, strength, and healing mechanism of a bisphenol‐A/adipic acid vitrimer system. The healing mechanism involved bisphenol‐A/adipic acid polymer chains undergoing transesterification exchange reactions. Post‐annealing, the healed vitrimer retained its intrinsic modulus and strength, demonstrating the reversible nature of damage accumulation through topological rearrangements in such systems. This healing strategy successfully addressed the irreversible nature of fatigue and achieved repeated reversal of fatigue‐induced damage in vitrimers and in vCFRP systems. The advancement of dynamic polymer composites capable of dependable self‐healing upon damage has the potential to revolutionize perceptions of material reliability, safety, maintenance, and life‐cycle expenses. These vCFRPs may lay the foundation for next‐generation materials in which natural aging and fatigue processes can be periodically counteracted, thereby guaranteeing secure and reliable long‐term performance.

#### 3.3.4 Pre‐Screening and Designing Catalysts

While the aforementioned MD studies offer valuable insights into tuning vitrimer properties by controlling bond‐exchange reactions, their accuracy level falls short of suggesting new materials or outlining how to experimentally control these reactions via catalysts. For example, these techniques can demonstrate the influence of bond swap energy barriers on various mechanical properties but cannot provide a viable method for controlling the energy barrier itself.^[^ ^21c^ ^]^ A common method to control the exchange reaction in vitrimers is through the use of catalysts, which results in different activation energies. To design new catalysts for vitrimers, it is important to understand the reaction mechanism, and DFT calculations can be a powerful tool to obtain thermodynamics and kinetics of the exchange reaction in vitrimers due to their high accuracy and ability to simulate chemical reactions at the electronic level. Such data can provide insights into the reaction mechanism and assist in pre‐screening and designing catalysts.

**Figure 11:**
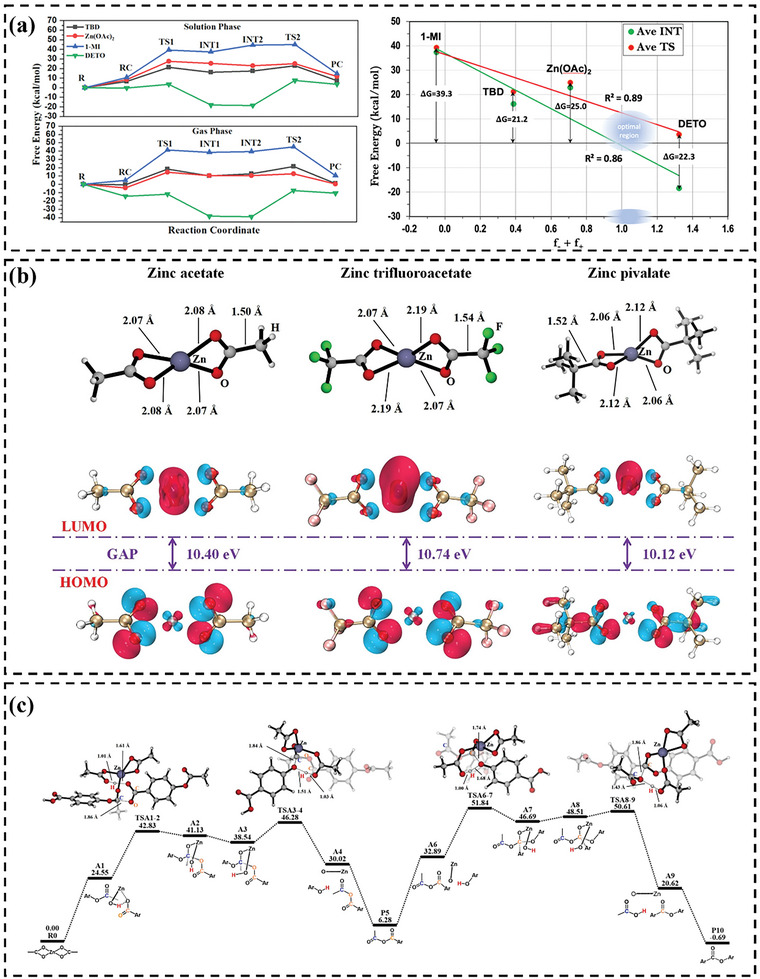
a) Reaction energy profiles for the transesterification catalyzed by 1‐MI (blue), TBD (black), Zn(OAc)_2_ (red), and DETO (green) in solution‐ and gas‐phase and solution‐phase transition‐state energies and intermediate energies plotted versus gas‐phase SN_2_ enhancement indices with linear fits for each energy series. Reproduced with permission.^[^ ^26^ ^]^ Copyright 2021, American Chemical Society. b) Optimized structure and the HOMO‐LUMO gaps of the chosen catalysts. c)Free energy surface of the polymerization of ABA catalyzed by zinc acetate. Reproduced with permission.^[^ ^25^ ^]^ Copyright 2022, Elsevier.

A common method to control the exchange reaction in vitrimers is through the use of catalysts,^[^ ^2^, ^99^, ^119^ ^]^ which result in different activation energies. DFT calculations can be used to estimate the free energies of relevant states along the reaction paths and obtain an ordering of the catalytic efficiencies by using the relevant energies of the identified barrier for the rate‐limiting step. For example, Bhusal et al.^[^ ^26^ ^]^ used DFT simulations to investigate the catalytic efficiency of the transesterification reaction mechanism with four different catalysts including triazabicyclodecene (TBD), zinc acetate (Zn(OAc)_2_), 1‐methylimidazole (1‐MI), and diethyltin oxide (DETO). Solvent effects were incorporated in the free‐energy results, and it was found that the efficiency followed the order TBD ≳ DETO ≳ Zn(OAc)_2_ > 1‐MI, which is consistent with experimental results (**Figure** **11**a). The rate‐limiting step was determined as the S_N_2 reaction,^[^ ^120^ ^]^ forming a tetrahedral intermediate, where the attacking alcohol and leaving alcohol remained simultaneously bonded. In addition, Fukui indices, which represented the change in partial charge on an atom due to removing an electron from the entire molecule, were calculated to examine the mechanism for lowering the barrier of the rate‐limiting step. The results showed that the sum of the base nucleophilicity index and the acid electrophilicity index of the bifunctional catalysts was highly correlated to the barrier energy of the rate‐limiting steps (Figure 11a). This finding suggests a plausible method for prescreening catalysts using computational simulations by identifying those with index values within a certain range.

In addition, DFT calculations can be useful in designing new catalysts which have been done for other polymer systems. For example, Shen et al.^[^ ^25^ ^]^ employed DFT calculations to explore the mechanism of the catalytic polymerization process involved in the synthesis of thermotropic liquid crystalline polymers using zinc(II) carboxylates as a catalyst (Figure 11b). DFT calculations revealed that the rate‐limiting step was the transesterification between phenol and anhydride acid. In the presence of zinc acetate, the energetically favored pathway was found, with a free energy barrier of 51.84 kcal mol^−1^ (Figure 11c), which was considerably lower than the energy barrier without a catalyst. This significant reduction of the energy barrier with zinc acetate was attributed to the role of zinc (II) carboxylate as a bridge to accept and donate protons. Moreover, modifications were made to the carboxylate group of zinc to observe the impact of the anion group on catalytic efficiency. It was discovered that zinc pivalate had higher catalytic efficiency, with a free energy barrier of 51.36 kcal mol^−1^ at the reaction temperature. Electronic analysts indicated that Zinc pivalate has the smallest HOMO‐LUMO gap (see Figure 11b) among the considered catalysts, indicating that its chemical activity was strengthened by the methyl substitution. We believe the same strategy can be applied to design catalysts for vitrimer materials.

#### 3.3.5 Chemical Modifications of Functional Groups

The industrial implementation of vitrimers may be hindered by the need for catalysts and the high catalyst loadings required in some cases.^[^ ^121^ ^]^ However, this challenge can be surmounted by employing exchange reactions that are not reliant on catalysts. Various associative exchange reactions have been utilized for the synthesis of vitrimers or vitrimer‐like materials, such as transcarbamoylation,^[^ ^20i^ ^]^ transesterification,^[^ ^2^, ^122^ ^]^ olefin metathesis,^[^ ^99b^ ^]^ silyl ethers,^[^ ^6^, ^123^ ^]^ or disulfide exchange.^[^ ^11^, ^20^, ^124^ ^]^

**Figure 12:**
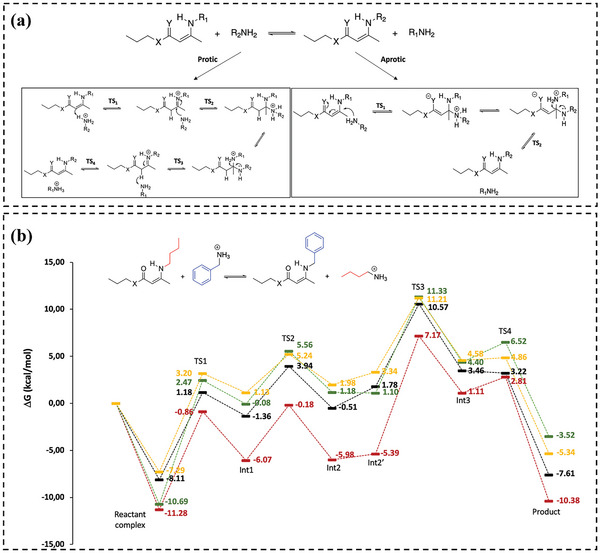
a) Proposed mechanisms of the exchange reaction in neutral and acidic conditions (protic, top) and in basic conditions or in the presence of Lewis acids (aprotic, bottom). b) Potential energy surface of the exchange reaction through the protic mechanism for different vinylogous acyl compounds. *X* = O (black line), S (yellow line), NH (red line), and CH_2_ (green line). Reproduced with permission.^[^ ^20k^ ^]^ Copyright 2022, Wiley.

Recently, the introduction of chemical groups covalently bonded in proximity to the dynamic bond has garnered significant attention as a means to influence the rate of the reaction. If these groups participate through weak interactions or steric effects, this phenomenon is known as internal catalysis.^[^ ^5^, ^10^, ^125^ ^]^ As a result, a judiciously designed chemical modification may lead to improved kinetics of the exchange reaction, without the need for external catalysts. Given the possibility of numerous chemical modifications, leveraging computational chemistry can accelerate the advancement of novel vitrimers by directing the design toward the most promising structures, rather than depending on the traditional trial‐and‐error methodology rooted in chemical intuition. High‐accuracy DFT calculations can be a powerful tool to expedite vitrimer development by directing the design of promising structures, replacing the conventional trial‐and‐error approach guided by chemical intuition. For example, Hamzehlou et al.^[^ ^20k^ ^]^ conducted a study to investigate the impact of chemical modifications on the exchange reaction of vinylogous acyl compounds using DFT calculations. Their study involved examining the proposed transamination exchange reaction mechanisms for vinylogous urethanes.^[^ ^7^, ^121^ ^]^ Specifically, they analyzed the transamination exchange reaction mechanisms of vinylogous urethanes by calculating the Gibbs free energy and obtaining the free potential energy surface. The study examined three different transamination mechanisms for vinylogous urethane with benzylamine: i) in neutral or acidic conditions (protic), ii) in basic conditions (aprotic), and iii) in the presence of a Lewis acid (**Figure** **12**a). The protic pathway displayed lower barriers, and the overall reaction path was less energetically demanding. The suggested mechanism for the protic pathway involved the initial protonation of the vinyl α‐carbon by a protonated free amine, generating an iminium cation. This step is identified as the slowest of the reaction, followed by the nucleophilic attack of a neutral free amine and the elimination of the original amino group in the vinylogous urethane. This leads to the transamination of the vinylogous acyl compounds. The reaction concludes with the newly formed free amine removing the proton from the iminium cation, yielding the new vinylogous urethane and free protonated amine. The influence of chemical modifications on the reaction is investigated by examining various vinylogous acyl derivatives and free amines within the protic pathway. The findings indicate that employing vinylogous urea (*X* = NH) results in the least energetic reaction path, owing to the stabilization of transition states and intermediates through hydrogen bonding interactions. (Figure 12b). In general, the use of reactants that promote hydrogen bonding interactions results in a stabilization of the reaction path and thereby may enhance the exchange reaction.

## 4 Conclusion and Future Perspectives

Vitrimer materials meet the environmental and performance requirements of today's world, representing another step toward a circular economy. In this review, we have summarized and given an overview of computational studies on vitrimers, which gives us a fundamental understanding of these materials and helps us guide the design of new materials with target properties. We highlight the advancements in DFT calculations and MD simulations of vitrimers (**Table** **2**).

**Table 2:** A summary of representative computational work for vitrimers.

Vitrimer materials	Method	Calculated property	Application	Ref.
Vinylogous vitrimers	DFT‐ωB97XD	Transamination exchange reaction mechanism	Controlled exchange kinetics by chemical modifications	[20k]
Schiff base vitrimers	DFT‐M06	Gibbs free energy	Mechanism of polyimine–metal complex vitrimers	[111]
Transesterification reaction vitrimers	DFT‐ωB97XD	HOMO‐LUMO, transesterification catalytic mechanism	Prescreening of potentially better catalysts	[26]
Vinylogous urethane vitrimers	DFT‐M06‐2X	Gibbs energy profile	Preparation of vinylogousurethane vitrimers	[7c]
Epoxy vitrimers	DFT‐BP86	Gibbs energy profile	Catalyst‐free transesterification vitrimers	[20a]
Enamine‐one bond based vitrimers	DFT‐B3LYP	Gibbs energy profile	Sealing and adhesion applications	[20c]
Diselenides vitrimers	DFT‐B3LYP	Bond dissociation energy	Design of smart materials	[105]
Fluorinated polyester vitrimers	DFT‐BP86	Gibbs energy profile	Catalyst‐free transesterification vitrimers	[20f]
Epoxy vitrimers	MD‐cutoff	Decomposition	Recycling	[103]
Disulfide vitrimers	MD‐cutoff	Self‐healing property	Vitrimer nanocomposites	[73a]
General vitrimers model	Hybrid MD/MC	Volumetric and rheological properties	Processing and end‐use applications	[21b]
Star‐shape vitrimers	CGMD	Swapping mechanism	Remolding	[45]
General vitrimers model	CGMD	Bond swap energy barrier	Tuning mechanical properties and self‐healing capability	[21c]
Thermo‐gelling vitrimers	Hybrid MD/MC	Entropy‐driven crosslinking	Biomedical applications	[21a]
General vitrimers model	Hybrid MD/MC	Creep and recovery	Creep associated applications	[21i]

Generally, MD/MD‐MC simulations can establish structure–dynamics–property relationships, which aids in understanding how the microstructure and dynamics of polymer chains influence the properties of interest. These simulations could offer insights into modulating the properties of vitrimers at the macroscale by tuning the material's structure or dynamics, including factors such as BERs rates, structural defects, and beyond. Key findings include the centrality of exchangeable bond lifetime in determining vitrimer rheology and dynamics; When deformation outpaces bond exchange, vitrimers resemble traditional thermosets, but at slower rates, they adopt a viscous character; The bond swap energy barrier profoundly influences chain dynamics, and optimal mechanical properties emerge at intermediate bond swap energy barrier values; Enhanced mobility, consequential to a reduced energy barrier, directly influences the stress–strain relation, favoring more pronounced deformation before fracture. Topological defects play an indispensable role in mediating exchange reactions and expediting stress relaxation. Moreover, altering bulk density facilitates the modulation of vitrimer fragility at *T*_g_, spanning from fragile to superstrong states, independent of the topology‐freezing transition temperature *T*_v_.

On the other hand, DFT calculations offer more detailed information on how to manipulate these chemical characteristics. Such calculations are particularly useful for studying reaction pathways and providing guidance on how to modify the chemical structure of the material at the atomic scale or to find the catalyst to improve the performance of vitrimer. Central discoveries from the contemporary DFT research encompass the following: Strategies to attenuate the bond exchange energy barrier have been elucidated. A prospective computational schema for the anticipatory screening of catalysts has been advanced. Further, insights derived from DFT calculations offer a methodical trajectory for the synthesis of vitrimers characterized by optimized exchange kinetics via targeted molecular modifications. As a result, the combination of MD and DFT is poised to play a vital role in the design of novel materials for vitrimers, significantly reducing development time and costs.

A prevailing challenge in vitrimer research pertains to the modulation of the equilibrium between mechanical stability and dynamic reversibility. In recent theoretical investigations, numerous methodologies have been proposed to optimize this balance. One such approach is the adjustment of bond exchange frequency within MD simulations. This adjustment elucidates the frequency with which covalent bonds dissociate and subsequently reform. An elevated frequency augments dynamic reversibility but may compromise mechanical stability. Another strategy is the manipulation of the bond exchange energy barrier (via catalyst or modified functional group). Elevating this energy barrier typically yields a vitrimer that is mechanically robust but exhibits diminished dynamic reversibility, and conversely, a lower barrier promotes reversibility at the potential expense of stability. Further, one might consider varying the density of crosslinking within the network; the crosslinking density can be tuned via the concentration of cross‐linkers. Generally, an augmented network density enhances mechanical stability while curtailing dynamic reversibility. Last, the selection of specific dynamic covalent chemistries for bond exchange presents another avenue for fine‐tuning the interplay between mechanical resilience and dynamic adaptability. Another challenge is the definition and detection of the *T*_v_. Originally defined as a threshold below which chemical exchanges become negligible, this concept is context‐dependent, rather than a strict phase transition. While the idea behind *T*_v_ is solid, treating it as an absolute or constant can be misleading. Its determination often requires extrapolations that can vary significantly. Although through MD simulations, two transition temperatures for a general vitrimers model have been predicted utilizing volumetric data, facilitating a deeper understanding of vitrimer behavior in the vicinity of *T*_g_ and *T*_v_ prediction of *T*_v_ for deterministic vitrimer materials has not been reported.

Despite the progress made in applying DFT calculations and MD simulations to the field of vitrimers, current research in this domain still presents numerous limitations.

### 4.1 Limited Scope

Most computational studies in the field focus on providing insights into the underlying mechanisms of vitrimers and supporting experimental results, rather than predicting new materials or exploring novel properties.

### 4.2 Model Complexity

The complexity of vitrimers, with their dynamic covalent networks and slow relaxation processes, can be challenging to model accurately, especially when simulating large‐scale systems and longtime scales.

### 4.3 Computational Cost

The cost of ab initio methods, such as DFT calculations, can be high when applied to large vitrimer systems, limiting the scope of the studies and potentially reducing their predictive capabilities.

### 4.4 Lack of Data

The scarcity of available experimental data on vitrimers can hinder the development of robust computational models and the validation of theoretical predictions. To overcome the challenges in computational studies of vitrimers and further advance the field, the following future directions are proposed.

#### 4.4.1 High‐Throughput Modeling

Implementing high‐throughput computational methods to screen and optimize vitrimer systems more efficiently, reducing the time and resources required for the discovery of new materials.

#### 4.4.2 Machine Learning

Utilizing machine learning techniques, such as developing descriptors or training models to predict properties and behaviors of vitrimers, can help in the discovery of new materials and the optimization of existing ones. The technique could also help to reduce the computational cost of screening new materials.

#### 4.4.3 Development of Advanced Computational Methods

Novel methods that can efficiently handle large systems and long timescales, as well as capture multi‐scale phenomena, are needed. Machine learning‐based approaches and enhanced sampling techniques could offer potential solutions.

#### 4.4.4 Improved Force Fields and Parameterization

Efforts should be dedicated to developing accurate force fields and parameters for vitrimer systems, accounting for their dynamic nature and diverse bond types.

#### 4.4.5 Investigation of Novel Dynamic Covalent Bonds and Vitrimer Systems

Computational studies should explore a broader range of dynamic covalent bonds and vitrimer systems to facilitate the discovery of new materials with unprecedented properties and applications.

#### 4.4.6 Multiscale Modeling

Combining various computational methods, from first‐principles calculations to CG simulations, can provide a more comprehensive understanding of vitrimer behavior across different length and time scales.

#### 4.4.7 Collaboration With Experimentalists

Strengthening the collaboration between computational and experimental researchers can improve the validation and applicability of computational models, as well as guide experimental efforts toward the most promising materials and systems.

The investigation of vitrimers has emerged as a captivating subject, poised to garner increasing interest as a result of burgeoning experimental and computational efforts. The challenges and opportunities associated with this field hinge on leveraging the predictive capabilities of DFT calculations and MD/MC simulations to unravel intricate and potentially unexplored response mechanisms. This approach also involves selecting suitable methodologies and examining the properties of novel materials. Consequently, it is imperative to integrate state‐of‐the‐art experimental strategies with theoretical research to elucidate the origins of vitrimer dynamics from molecular and atomic perspectives. Such insights will guide the development of superior materials and foster the broader implementation of vitrimer materials in industrial and everyday applications.

## Conflict of Interest

The authors declare no conflict of interest.
